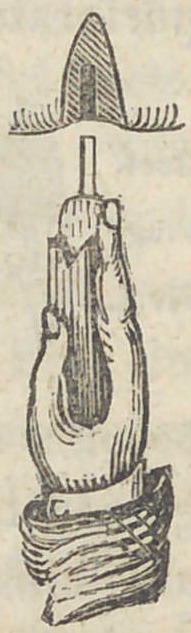# Correspondence

**Published:** 1861-01

**Authors:** 


### Correspondence.

[The following letters, which we take the liberty of publishing, from the pen of Dr. I. J. Greenwood, of New York, contain many particulars and items of interest in regard to the early history of the profession in the United States. These go as far back as to the time of his grandfather, Horace Greenwood, of Boston, Massachusetts—as early as 1750.

These letters give us some idea of what dentistry was in those days, and how it progressed—how it moved on step by step. The first of these letters was elicited by our inquiry of Dr. Greenwood, as to who was the first that used and introduced to the profession plaster of Paris models for forming plates and fitting teeth. This has been clearly answered, as well as many other points of which we were ignorant, and about which we feel much interest.

There is a biographical sketch of Mr. John Greenwood published in the first volume of the *American Journal of Dental Science.* It, however, has reference to him more in other particulars than as a dentist. These letters will repay the most careful perusal.—ED.]

---

NEW YORK, November 3, 1860.

DR. TAFT.—*Dear Sir:*

In a letter received from you to me under date of October 27, 1860, you request me to inform you of the first use of plaster of Paris models for the forming of plates to the same to receive artificial teeth, as far as I am informed.

My grandfather, Isaac Greenwood, of Boston, Massachusetts, practiced the making of artificial teeth there many years previous to the Revolution, but from what I can gain as to information, he never used plaster of Paris to make models of, for either plate or bone work; he made his teeth out of the sea-horse tooth (hippopotamus), and used merely a beese-wax mould, as did my father, John Greenwood, who practiced in New York from 1790 to 1820. He was the particular dentist of General George Washington, as expressed in a letter from him to my father, dated Mount Vernon, 6th January, 1799, thus: "If you should remove to Connecticut, I should be glad to be advised of it and to what place, as I shall always prefer your services to that of any other in the line of your present profession."

There is a pair of false jaws with human teeth on, *now* in the head of President Washington, "in his tomb at Mount Vernon," made by my father, John Greenwood, in 1799, and they were made with bone gums—I think of the *elephant's tooth* "ivory," and made from moulds of beeswax. My brother, Mr. Clarke Greenwood, deceased, and myself did not use plaster of Paris until about 1820, and I think it was through my own suggestion. "We hardened them by dipping the plaster moulds into boiled linseed oil, and let them dry." Before that time white and yellow bees wax was much in use for plate and bone work, even for half and whole sets of teeth. I never had a set returned to me on account of the fit. And I think I was the first in New York who set natural human teeth upon bone gums and colored the gums to life, after those made for General George Washington by my father in 1799. I was the first, about the year 1823, to use the *steel* burr in a lathe which I invented, and had made for me in New York, by a Mr. Morgan, a Scotchman, for excavating the cavity for the gums to rest in, in parts of, and whole sets of teeth. And I claim the first use and discovery of wooden pivots, to be used in bone or mineral teeth. And the first mineral teeth seen by me were brought to the country by Colonel Aaron Burr, whose dentist my father was, and presented to him by the Colonel. They were shaped thus, with platina pivots attached, taken and imbedded in the material, to solder them to plates, etc. The first I ever saw in New York and made in the United States, were made after the same plan, but thicker, thus:

In the first years of my practice metallic pivots to teeth were used, and screwed into the material of bone or human teeth (or sea-horse, sheep or oxen), and cotton was wrapped round the metal pivots to keep them in the sockets of the roots, which, when decomposed, would give an offensive odor; and with me it was a great point to endeavor to find a remedy to prevent these bad effects, and to keep the teeth sweet and clean as possible, which I soon was enabled to remedy, thus.

About 1825, I was operating to fasten a single tooth for an English gentleman, the root of which had been "*bushed*" with bass wood or soft maple, to enable the pivot of gold to retain its place firmly, for which, after being well fitted to the cavity of the root, the wooden plug had been perforated that the gold pivot might rest in it firmly, thus.

Root.
Plug,
Pivot.

Finding that the wood answered the purpose well, and that very little *odor* or smell arose from the wooden plug; the idea suggested itself to me that *wood* might be a proper medium to use for pivots to teeth instead of metal, and if I could *procure* a wood that would answer my purpose, I should have gained the point *desired*. After considering, I concluded to use hickory wood, well seasoned and dried, and *straight grained* (white part), and I had the good fortune to procure some straight grained white, which had been used by a baker in the making of bread, some ten years in use (the same as spokes of wheels are made of and ax handles); fiber straight, long and tough. I got this from a cartwright or wagon-maker, and it lasted me all the time I followed the profession until I retired in 1841. The way I used it was thus : I first prepared the tooth to fit the place or *arch* of the root on which it was to rest, as seen by the following illustration :

Root and Gum.

Toot and Pivot.

Having fitted the tooth with a pivot made of softer wood, *good white or Georgian pine*, straight grained, I prepared my proper pivot of hickory thus: The drill which made the cavity for the pivot in the tooth was made to suit with the cavity in a draw-plate, through which I passed the hickory pivot, having first cut it with a knife and filed it to suit the hole in the draw-plate. I then drew it the same size to fit the hole in the tooth, then, with a hand-drill of the same dimensions as the pivot, I made the hole to correspond with that in the root; and when the tooth was finished and ready to receive the hickory pivot, I fastened the same in the tooth, and cut it off somewhat *less* than the depth of the hole in the root and then pressed it up into the root of the tooth, where, *if well adapted*, it was almost impossible to pull it out. For the purpose of steadying the tooth and pressing it up with the pivots attached to it, I made an instrument thus, of hickory, to be used in this manner.

The instrument caught the end of the tooth (in the crotchet) and the other end resting on the palm of the hand, I pressed the pivot with the tooth attached into the root cavity, and, *if well adapted*, it always answered well. Indeed they answered so well that I was often forced, when they broke off from friction wearing the wooden pivot off, to redrill the cavity, with the end of the pivot in it, out again, to replace it by another wooden pivot. After this, except in extraordinary cases, I never used metallic pivots for single teeth, and the mouth was sweet and clean by those who were careful to brush their teeth regularly.

My father was the first to use the "*foot-drill*," and he made it himself from an old spinning-wheel of my grandmother's; and, since his death, I myself used it, the same one, altogether in my practice for twenty years, and have it yet. I never had seen one before, and I know the hand bow-drill was always used before. I never used the hand bow-drill to perforate the roots of teeth for pivots, etc., nor in any way, but a drill instrument with a spear-shaped point, gauged for the depth of the pivot, to drill the roots to receive the wooden or metallic pivot. But to make the hole to receive the pivot in the tooth, I always used the *foot-drill*. And in drilling pieces of bone or ivory, I could, with the drill made of the finest needles, meet the drill-hole an inch apart.

The drill for the hole for *pivot* of wood in the false tooth and for the hole in the root, should fit exact with the hole in the draw plate you drive the pivot of wood through.

I was the first dentist who had mineral teeth prepared with holes in them to receive *wooden pivots*.

Hoping the above may prove of use to you, I am, dear sir,

Yours, respectfully,

Isaac John Greenwood, D. D. S.

---

New York, November 14, 1860.

Dr. J. Taft.—*Dear Sir;*

In your letter to me under date of the 9th of November, you request me to give you some information of the early history of dentistry in the United States; and how far my relations were concerned before myself in the profession; and in what manner they gained their information relative to the science? From what I have been enabled to gather from my father and my relatives, all I can inform you of is, that my grandfather, Isaac Greenwood, who was born and lived at Boston, and was the first practitioner in dentistry in the family (if it may be called practicing dentistry), was the remaining son of Isaac Greenwood, of Boston, Professor of Natural Philosophy and Mathematics in Harvard College, Cambridge, Massachusetts; and he was the only son who studied mathematics as his future occupation and practice, to the end that he might be a mathematical instrument maker, etc. He was, about the year 1750, a mathematical instrument maker, and ivory and wood turner, umbrella manufacturer and dentist. He followed all these professions at the same time, and made the first electrical machine for Benjamin Franklin; my uncle Isaac told me so, and he was apprentice with his father and eldest son.

Where my grandfather procured his information in dentistry, it is impossible for me to say, and I presume his practice was confined to the mechanical portion; although in his portrait (large as life), taken some time after this, he is depicted with his left hand and arm resting upon an open volume of *Hunter's Treatise upon the Human Teeth*, which portrait and the treatise I have in my possession. The specimens of the teeth then made by him are very *rude*, imperfect and ill-shaped, merely a piece of sea-horse tooth formed to suit the space to be filled up, where the natural teeth were wanting, and a separation or slit made with a file (the enamel of the piece of sea-horse being ground white on a grindstone), with no manner of an attempt at formation or imitation of natural teeth. They were not, in some instances, arched on the top, and were fastened on with thread or wire, silver or gold.

I never saw springs, *flat* or *spiral*, used or having been used, until my father, John Greenwood, used them in his practice, which commenced eleven years before I was born, 1795, and about the year 1784, in New York city.

My grandfather, Isaac Greenwood, had four sons and one daughter. The sons were named, Isaac, John, William Pitt and Clark, and what information they gained they procured from their father at Boston and in his shop, as they were all with him there employed. Being with him in his occupation at Boston, what information they gained in dentistry they got from him in his practice I presume.

Isaac Greenwood, my eldest uncle, practiced dentistry in all its branches, and was an excellent mechanical dentist, in Providence, Rhode Island, many years, until he came to New York. He likewise kept a hardware store, etc., there. The second son was my father, John Greenwood, the accepted and private dentist of General George Washington. He never gained much information of the art of dentistry. He studied or was an apprentice with his father's brother, John Greenwood, who lived at Portland, State of Maine now. He was a cabinet maker, merchant and ship-owner there. His brick house there was the only one uninjured when the British fired upon the town in the Revolution, and it stood upon the present site of the White Marble Hotel, not yet finished, on the Main street, now in Portland, which was built thus far in expectation of the *Great Eastern* stopping at that place, etc.

My father never gained much information of the art, as he joined the Revolutionary army at fourteen years of age, previous to the battle of Lexington, and was at the battle of Bunker's Hill, etc. But after the peace he practiced as a mathematical instrument maker, and quit that business to follow that, to him, more profitable, mechanical and surgical dentistry, etc, which he followed until his death.

The third son, William Pitt Greenwood, was instructed by his father at Boston. He was a perfect master of the profession, and died wealthy from the practice of it at Boston.

The fourth and youngest son learned the profession of his father, but never practiced in New York city any further than to supply those deficiencies which his own particular case might require. He was a very neat workman, and was a mathematical instrument maker in Front street, city of New York, near the Tontine Coffee-house.

The daughter's son, Mr. George Henry Gay, of Dedham,

Massachusetts, studied for a doctor and surgeon, and for a dentist, both surgeon and mechanical, in Boston, where he acquired a great reputation in the practice of dentistry, and particularly in repairing and replacing *palatial deformities.* All the above named persons are now dead.

My uncle, William Pitt Greenwood, and my cousin, George Henry Gay, M. D., were both well conversant with the inserting of mineral teeth, and manufactured them in their practice in Boston.

Two years before the practice of my father in New York. the following advertisement occurs in Rivington's *Royal Gazette*, of New York, for August 24th, 28th and 31st, 1782: "TEETH.—Any person who is willing to dispose of his front teeth, may hear of a purchaser by applying to No. 28 Maiden Lane, for which a generous price will be given.

"N. B. Four guineas will be given for every tooth."

The above teeth were, no doubt, required to be replanted into the cavities of the alveolar process, to take root there and supply the loss of the ones to be eradicated for the operation.

I have in my possession a skull with an under jaw, left side bicuspides, which has been *inserted* or engrafted in this way and taken root, or attached itself to the process. This skull was brought from Paris by my father, John Greenwood, about 1806, where he went to procure a keg of natural human teeth.

That dentistry was practiced *prior* to the Revolution in the Provinces of America, we learn from the following. The *Constitutional Gazette* of April 24th, 1776, Boston, after stating that the body of General Joseph Warren had been re-interred at Boston on the 8th, states: "The General's remains were found on the fourth instant, about three feet under ground, on Bunker Hill. They were known by two *artificial teeth*, fastened by gold wire," etc.

If the above information can be pleasing to you, or as information to any one in the profession, you can make what use you please of it, as you may depend upon anything which I write you upon the subject, as far as I am informed.

With respect, I am, dear sir, yours, etc.,

ISAAC J. GREENWOOD,

No. 142 West 14th street, City of New York.

---

MESSRS. EDITORS:—I have but little to communicate at this time; nothing of special interest occurring here just now worth noting.

The class in attendance at the dental school here is the largest they have ever had, numbering between sixty-five and seventy. What the extent of the class at Baltimore is I am unable to say, but presume a fair one.

As a collateral matter I give you a specimen of the inflated estimate they place upon their manufactures in New York, and which possibly indicates the manner of estimating their population as well as manufactures.

In the "Merchants' and Bankers' Register" for 1860, which gives the State (New York) census for 1855, I find under the head of King's county, Brooklyn, the following: "*Dentists' gold manufacturers, one establishment, employing two hands, with two hundred and fifty dollars' worth of machinery, and consuming ten thousand dollars worth of raw material, produces one hundred thousand dollars' worth of manufactured articles.*"

This is surely a very remarkable business. See, the labor of two hands, with the consumption of ten thousand dollars, make *one hundred thousand dollars product.*

Brooklyn is evidently the place for dentists' gold manufacturers to make money, and render their capital and labor *largely* remunerative; but unfortunately these figures can not be relied upon, for they carry their refutation upon their face.

The last (October) number of the Baltimore Journal is a good one, especially in the variety of the contents, and shows the work of a new or fresh hand in this, in its management, as well in the apparent forgetfulness or ignorance of the current dental periodical literature of the day, as is evidenced by translating and publishing, from a German periodical, an article which originated in our own language, and in one of our dental journals, months ago; but this journal always had a mania for translations, many of which were greatly dignified thereby, and in some instances the language in which they appeared constituted their sole merit. But as I have already said, the number is a good one and exhibits a degree of vitality and sprightliness, which, if kept up, will entitle it to the claim of a *live* journal. A little *less allopathy* (which seems to require constant defense and a persistent abuse of all other systems) and *more dentistry*, would be a decided improvement in its pages, and be more in keeping with its title. It has no more earnest friend and well wisher than the writer, not only because of the good it has done but for the good it may still do, if it but keep pace with the progress of the profession. Yours, etc., O. U. C.

*Philadelphia, December* 22, 1860.

---

The Executive Committee of the Kentucky State Dental Assocition has selected the subjoined list ot subjects for discussion at the ensuing annual meeting to be held in the city of Louisville, on the second Tuesday of April, 1861.

1. Treatment of Temporary Teeth.
2. Causes of Caries and Prophylactic Treatment.
3. Filling Teeth.
4. Extraction of Teeth.
5. Tobacco—its Effects upon the Health of the Mouth.
6. Artificial Denture.
7. Miscellaneous.

The committee would suggest to members of the profession designing to attend the meeting of the Association, the propriety of their coming prepared to illustrate either, by drawings or with instruments, anything new or peculiar in their practice. J. W. GRANT, *Chairman.*

## Figures and Tables

**Figure f1:**
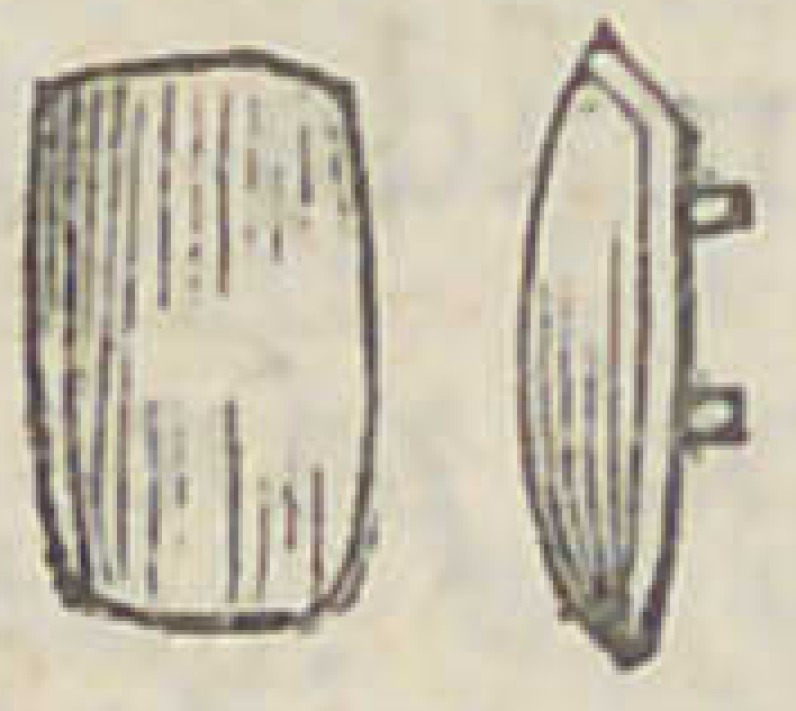


**Figure f2:**
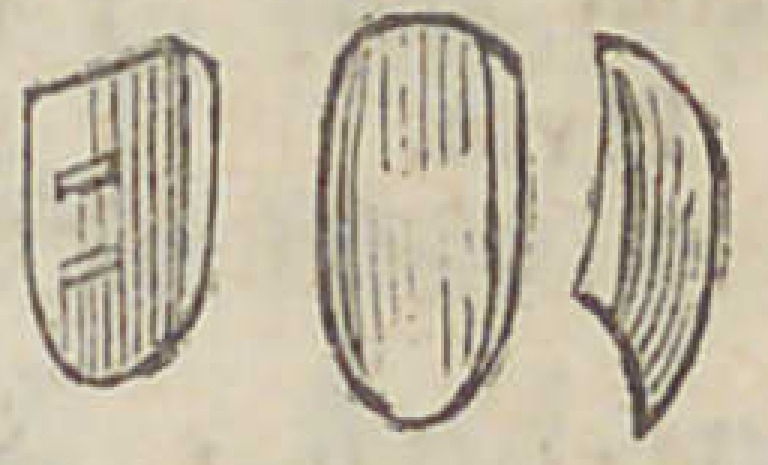


**Figure f3:**
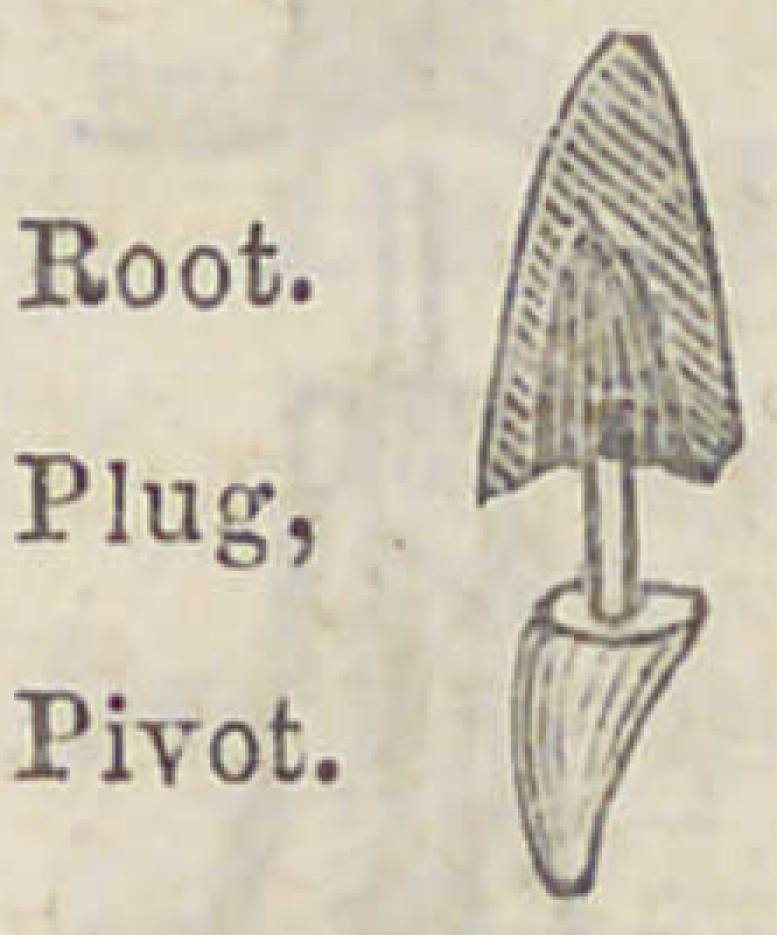


**Figure f4:**
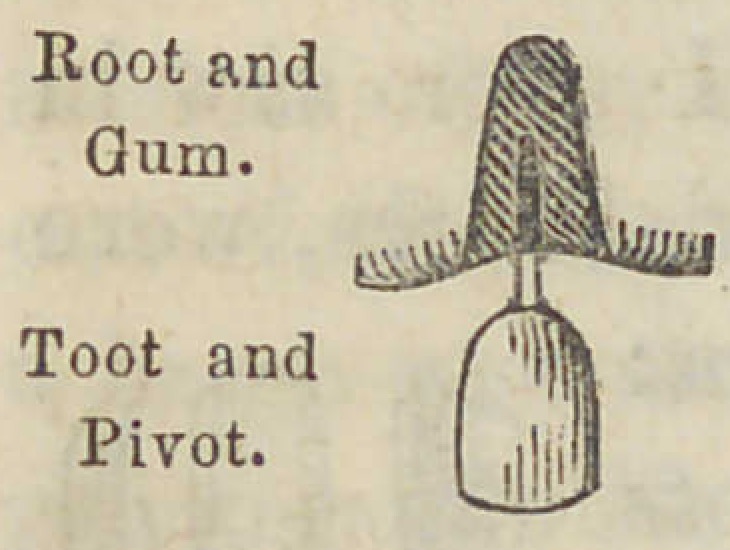


**Figure f5:**
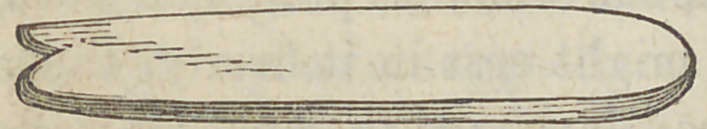


**Figure f6:**